# Supplementary material for: Evaluation of the Two-Point Ultrasound-Guided Transversus Abdominis Plane Block for Laparoscopic Canine Ovariectomy
Source: Animals (Basel). 2022 Dec 15;12(24):3556. doi: 10.3390/ani12243556 (PMC9774418; doi:10.3390/ani12243556)
Supplement: Supplementary file 1 [file animals-12-03556-s001.zip › Supplementary table S1.pdf]

Table S1. Values of end-tidal isoflurane concentration (EtISO), heart rate (HR), and mean invasive blood pressure (MAP) at the beginning of the surgery (Preop), after the resection of the first ovary (O1), after the resection of the second ovary (O2), and at surgical closure after pneumoperitoneum deflation (Postop).

	**CONTROL GROUP**				**TAP GROUP**				**Adjusted *p***		
	Preop	O1	O2	Postop	Preop	O1	O2	Postop	Over time	Between groups	Interaction
EtISO **(%)**	0.73±0.14[a]	1.17±0.16[b]*	1.18±0.19[b]*	0.89±0.10[c]	0.79±0.12[a]	0.91±0.14[b]	0.88±0.09[b]	0.85±0.07[a]	0.0001	0.0001	0.0001
**HR (beats/minute)**	53.12±9.13[a]	71.77±17.43[b]	73.88±16.73[b]	73.54±15.76[b]	57.38±16.44[a]	74.31±21.88[b]	76.96±32.68[b]	68.35±15.56[a]	0.0001	0.59	0.54
**MAP (mmHg)**	86.27±10.31	86.19±15.43	89.12±17.12	86.65±11.67	92.46±14.55	89.35±12.78	95.81±16.65	87.38±14.12	0.14	0.06	0.76

Data are shown as mean± SD. Within each group and in the same row, data with different superscript letters show significant differences between time points ($P < 0.05$). The asterisk indicates a significant difference compared with the same variable and time point in the TAP group. *p <0.0001.
