# Supplementary material for: Evaluation of the Two-Point Ultrasound-Guided Transversus Abdominis Plane Block for Laparoscopic Canine Ovariectomy
Source: Animals (Basel). 2022 Dec 15;12(24):3556. doi: 10.3390/ani12243556 (PMC9774418; doi:10.3390/ani12243556)
Supplement: Supplementary file 1 [file animals-12-03556-s001.zip › Supplementary table S2.pdf]

Table S2. Pain scores' values at different time points according to the Glasgow Composite Pain Measurement Scale (CMPS), the Melbourne Pain Scale (MPS), and the Colorado Pain Scale (CPS).

		Preop	T1	T2	T3	*p*
CMPS	CONTROL	0 (0-0)^{a}	1 (1-7)^{b*}	1 (0-7)^{c*}	0 (0-2)^{a**}	0.0001
	TAP GROUP	0 (0-0)^{a}	0 (0-2)^{b}	0 (0-0)^{a}	0 (0-0)^{a}	0.0001
MPS	CONTROL	0 (0-0)^{a}	2 (0-5)^{b*}	0.5 (0-5)^{c*}	0 (0-3)^{d***}	0.0001
	TAP GROUP	0 (0-0)^{a}	0 (0-2)^{b}	0 (0-0)^{a}	0 (0-0)^{a}	0.0001
CPS	CONTROL	0 (0-0)^{a}	2 (1-3)^{b*}	1 (1-2)^{c**}	1 (1-1)^{d}	0.0001
	TAP GROUP	0 (0-0)^{a}	1 (1-1)^{b}	1 (1-1)^{b}	1 (1-1)^{b}	0.0001

Data are shown as median and range (min-max). Within each group and in the same row, data with different superscript letters show significant differences between time points ($P < 0.05$). The asterisks indicate significant differences with the TAP group for the same variable and time point. * p =0.0001; ** p =0.039; ***p = 0.005.
