# Supplementary figures and images for: Evaluation of the Two-Point Ultrasound-Guided Transversus Abdominis Plane Block for Laparoscopic Canine Ovariectomy
Source: Animals (Basel). 2022 Dec 15;12(24):3556. doi: 10.3390/ani12243556 (PMC9774418; doi:10.3390/ani12243556)

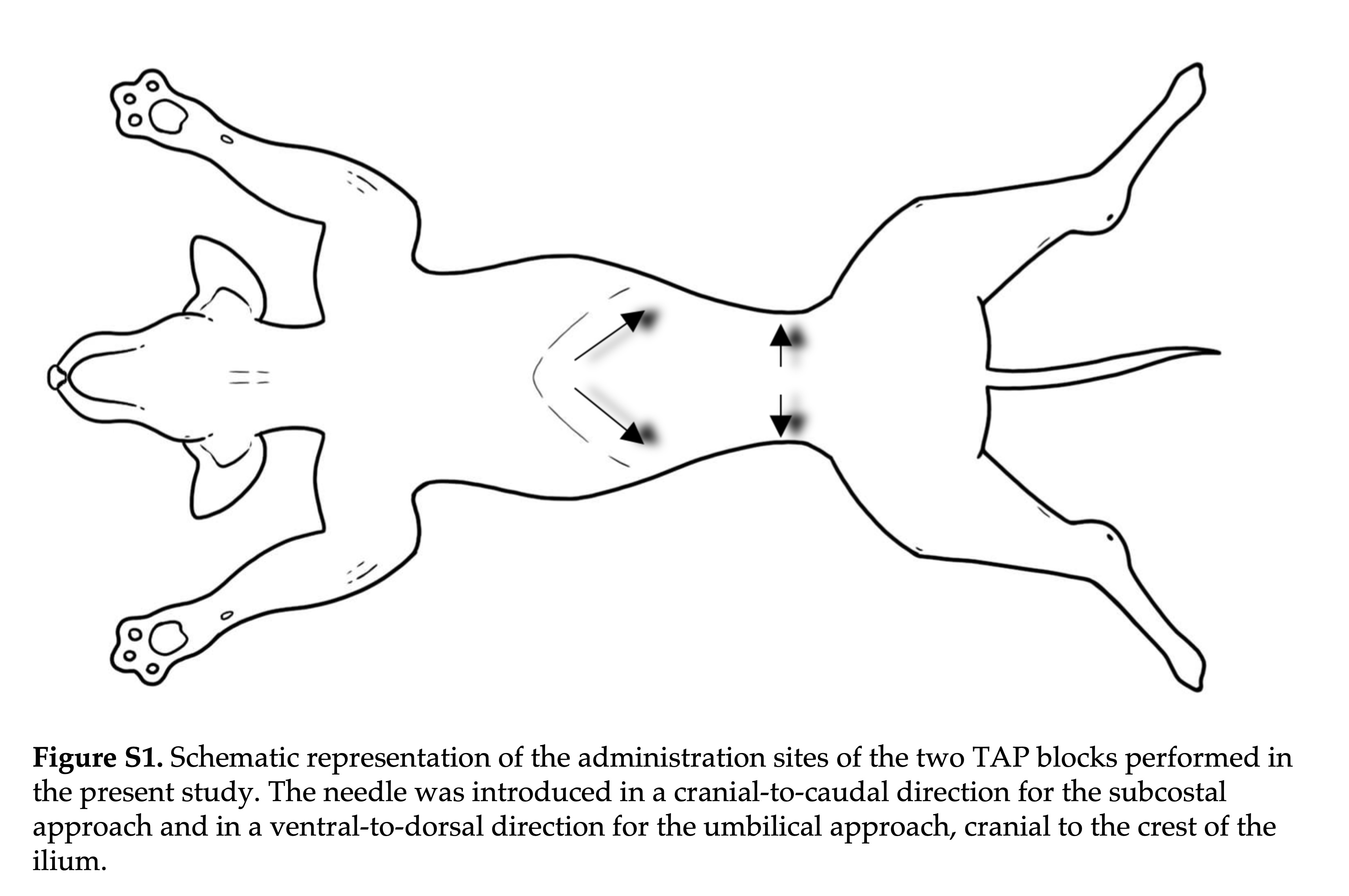

Supplement: Supplementary file 1 [file animals-12-03556-s001.zip › Figure S1.jpg]

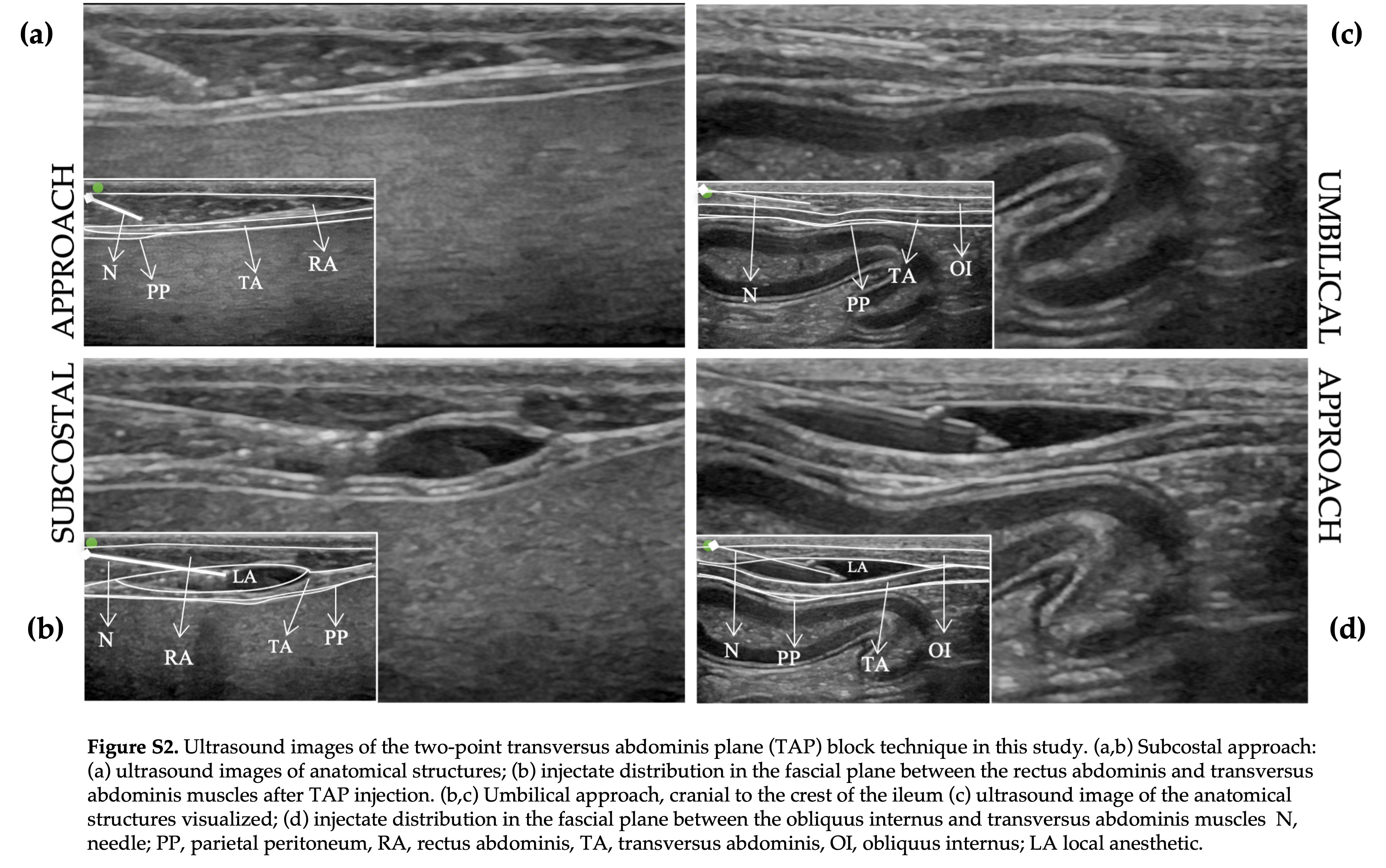

Supplement: Supplementary file 1 [file animals-12-03556-s001.zip › Figure S2.jpg]
